# Supplementary material for: Nuclear actin structure regulates chromatin accessibility
Source: Nat Commun. 2024 May 15;15:4095. doi: 10.1038/s41467-024-48580-y (PMC11096319; doi:10.1038/s41467-024-48580-y)
Supplement: Supplementary file 1 — Supplementary Information [file 41467_2024_48580_MOESM1_ESM.pdf]

Sen et al

Nuclear actin structure regulates chromatin accessibility

Supplemental Figures

Figure 1a/b: Actin structure in cells

Figure 2: Osteogenesis and adipogenesis are modulated by actin.

Figure 3: Heatmaps of known adipogenic and osteogenic markers at 24 h.

Figure 4a/b: Actin structure is phenotypically distinguishable during differentiation.

Figure 5: Chromatin accessibility is altered by CK666 at 4 hours in bone marrow mesenchymal stem cells.

Figure 6: Chromatin accessibility is altered by CK666 at 4 hours in the NIH3T3 embryonic fibroblast cell line.

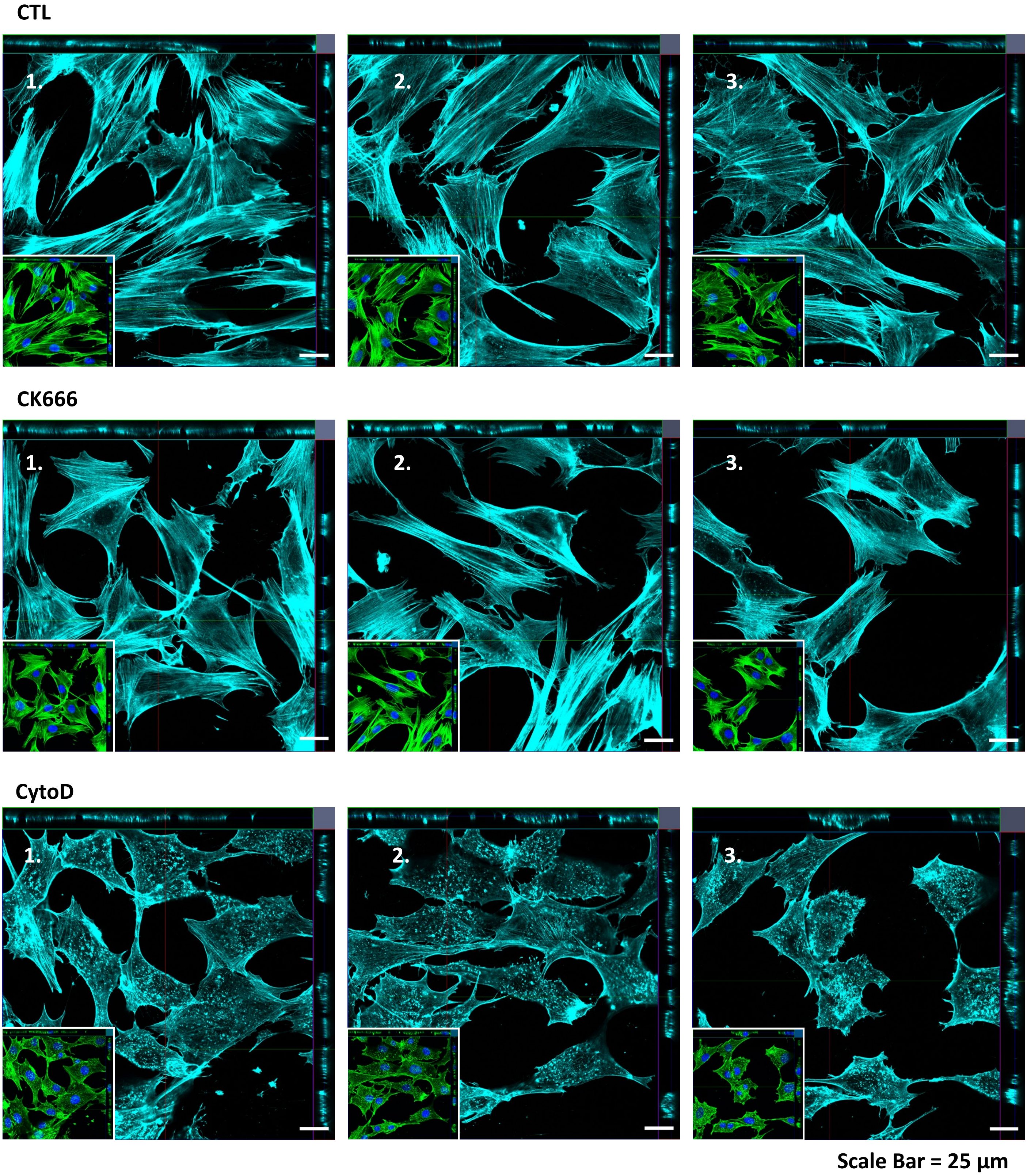

Supplementary Fig 1a. Low power micrography of cells treated with actin disrupting agents stained for F-actin (phalloidin) and nucleus (NucBlue) show phenotypically distinguishable cells between conditions. A single cell in each well is used for higher power resolution of nuclear structures.

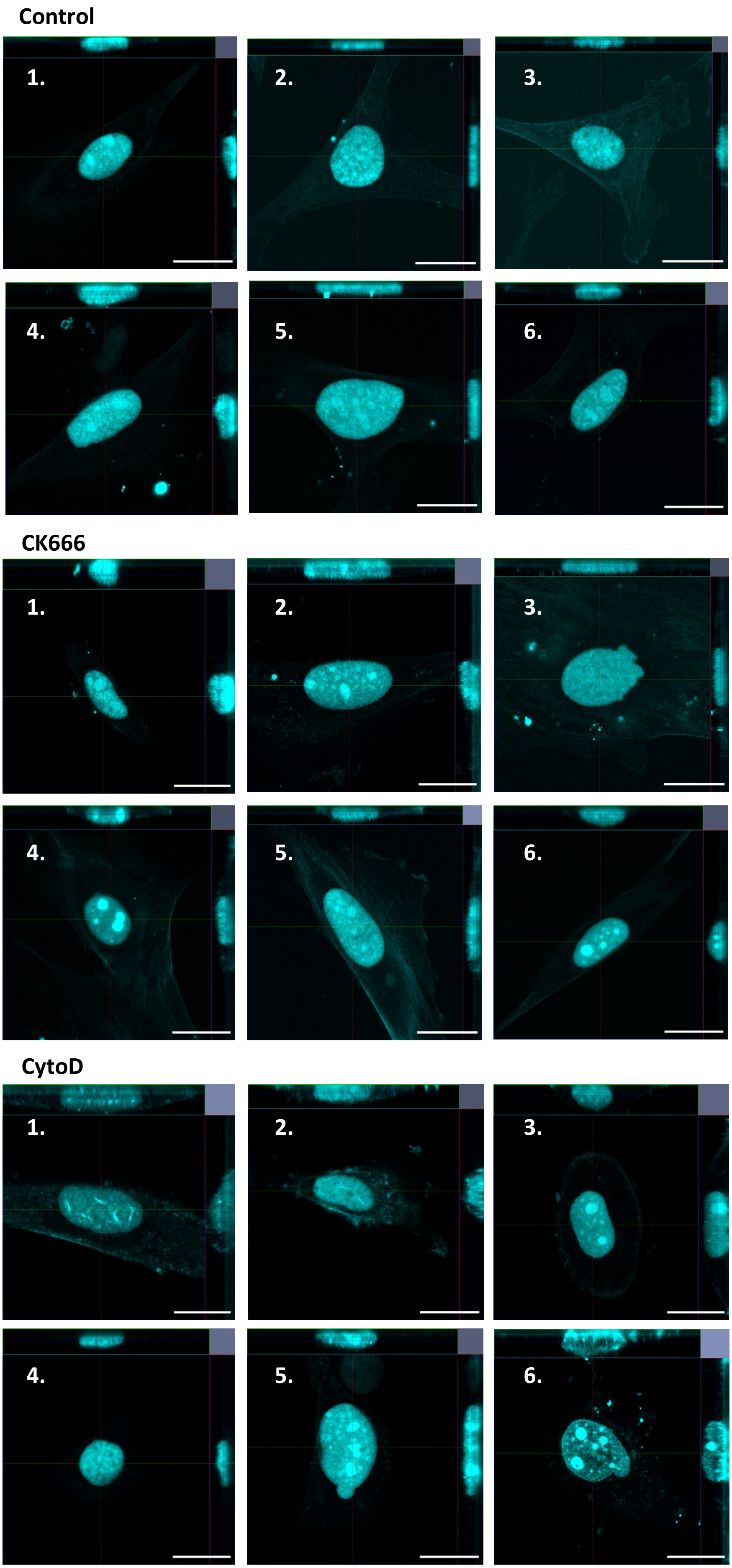

Supplementary Fig 1b. Confocal micrographs of nuclei after transfection with nuclear actin chromobody (control, +CK666, CytoD), 6 further examples.

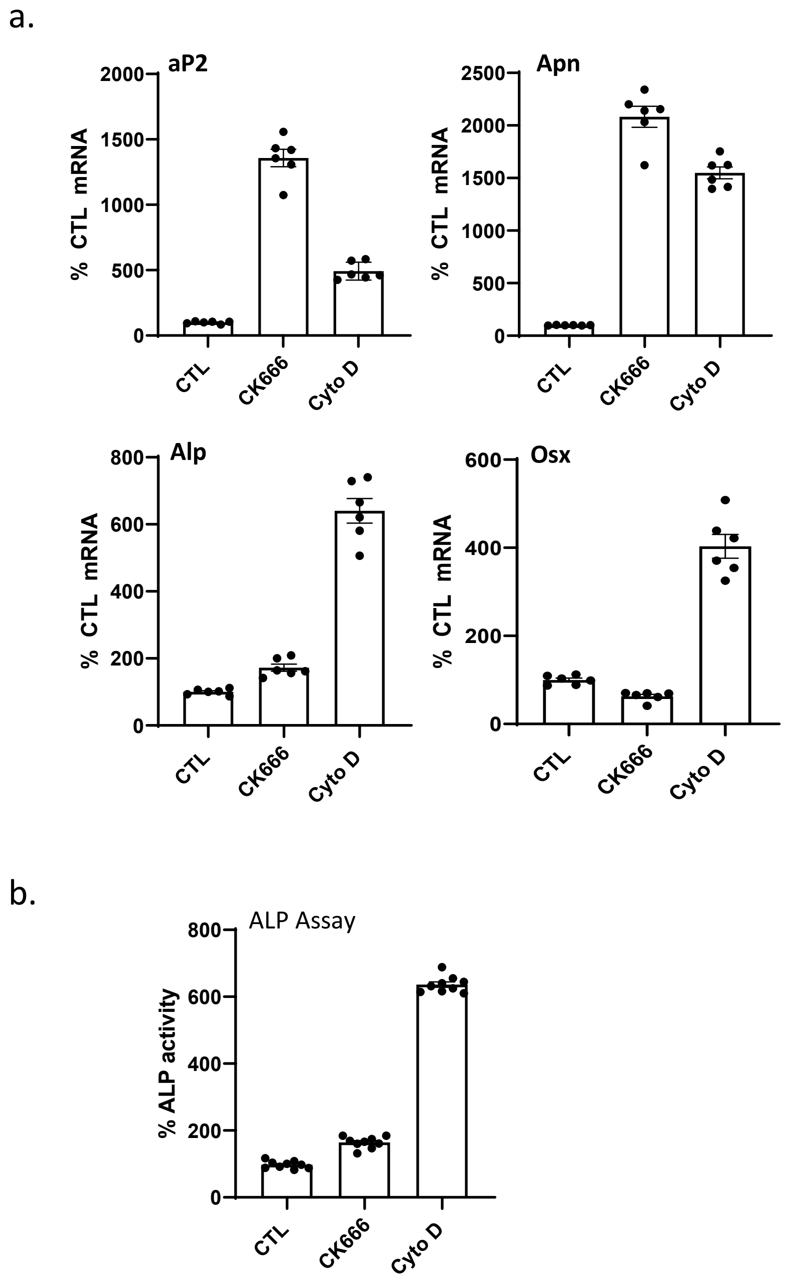

Supplementary Fig 2. Osteogenesis and adipogenesis are modulated by actin toolbox. Cultures were treated continuously with control, CK666 or CytoD for 3 days. (a) RT-PCR for adipogenic genes (aP2, adiponectin) or osteogenesis (Alkaline phosphatase, osterix) shows expected 3 days outcomes for disruptions of actin polymerization. (b) Alkaline phosphatase assay shows that CytoD treated cells generate alkaline phosphatase protein.

Adipogenic	Control 1	Control 2	Control 3	CK666 1	CK666 2	CK666 3	CytoD 1	CytoD 2	CytoD 3
Pparg	-0.88	-1.31	-1.39	1.64	0.36	0.49	0.12	0.39	0.59
Cd36	-1.31	-0.91	-1.60	0.59	0.96	1.02	0.37	0.32	0.57
Lipe	-1.23	-1.08	-0.84	-0.21	-0.25	-0.05	1.05	1.16	1.44
Ascl1	-0.33	-0.33	-0.33	-0.33	2.67	-0.33	-0.33	-0.33	-0.33
Adipoq	-0.82	-1.44	-1.15	0.38	0.07	-0.34	1.11	0.96	1.22
Adhfe1	-1.23	-1.31	-1.42	0.55	0.42	0.55	0.83	0.77	0.83
Nfia	-0.11	-1.08	0.10	-1.60	-0.57	-0.05	0.69	1.26	1.36
Adipor2	0.56	0.60	0.97	-2.36	-0.29	-0.53	0.54	0.18	0.33
Cidec	-0.83	-1.30	-0.88	1.68	-0.27	-0.51	0.38	0.80	0.94
Plin1	-1.02	-1.21	-1.61	0.53	0.75	0.17	0.81	0.50	1.07
Cebpa	-0.52	-0.85	-0.72	-0.26	-0.89	-0.68	1.12	1.29	1.50
Fabp4	-1.37	-1.16	-1.24	1.31	0.03	0.43	0.60	0.55	0.83

Osteogenic	Control 1	Control 2	Control 3	CK666 1	CK666 2	CK666 3	CytoD 1	CytoD 2	CytoD 3
Rgs2	-0.71	-0.43	-0.65	-0.57	-0.88	-0.72	1.31	1.27	1.39
Nr4a3	0.64	1.54	0.73	0.07	-0.78	0.01	-2.00	0.05	-0.27
Prkaa2	-0.74	-0.48	-1.09	0.69	1.64	1.43	-0.19	-0.64	-0.63
Dlx5	-0.12	0.11	0.30	-0.83	-1.73	-0.86	1.25	0.68	1.20
Fgfr2	-0.27	-1.07	-0.99	-0.73	-0.13	-0.61	1.06	1.36	1.38
Atoh8	-0.45	-0.79	-0.93	0.58	-1.13	-0.78	1.05	1.22	1.22
Runx2	-0.14	0.88	0.72	-2.51	0.42	-0.11	0.29	0.35	0.09
Ccno	-0.36	-0.96	-1.48	0.76	-0.76	0.08	0.65	1.75	0.33
Slfn5	-1.37	-0.01	-1.15	-0.26	1.88	-0.59	0.32	0.69	0.50
Sp7	-1.31	-0.89	-0.23	-0.56	0.78	-0.60	0.67	1.91	0.23
Bglap	-1.28	-1.28	-1.28	0.64	0.88	0.49	0.06	1.09	0.69

Supplementary Fig 3. Heatmaps of known adipogenic and osteogenic markers at 24 h. Shown are Z-scores of gene expression. For adipogenic genes, both CK666 and CytoD show increase at 24 h, which in the case of CK666 continues toward full expression at 72 h. For osteogenic genes, CytoD shows increase at 24 h, which continues.

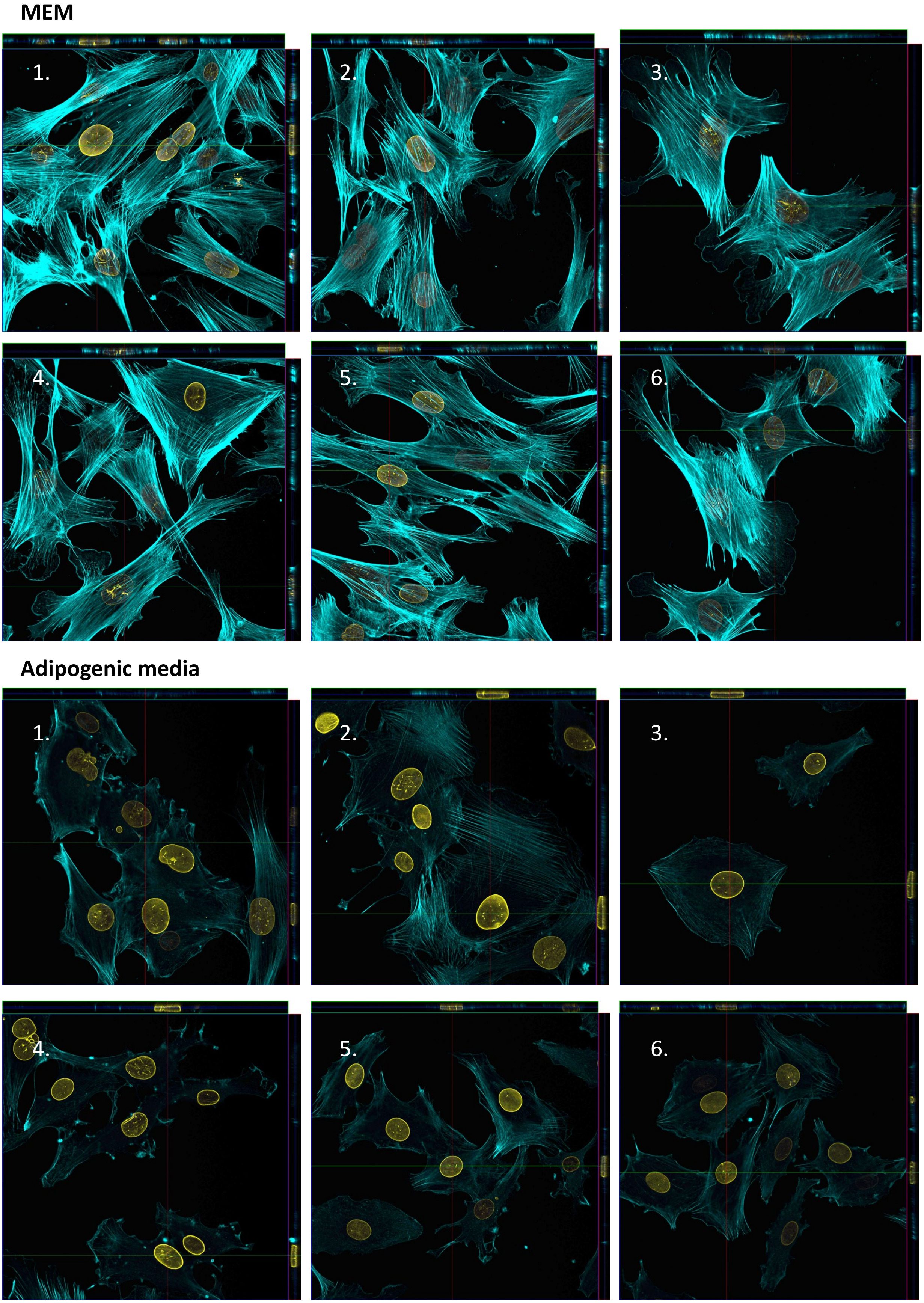

Supplementary Fig 4a Confocal microscopy of F-actin and Arp4 comparing control (MEM) and cells treated with adipogenic differentiating medium. Adipocytes show reduced F-actin structure compared to control cells. Arp4, restricted to the flat nucleus, is similarly enriched in both phenotypes at the inner nuclear membranes.

MEM

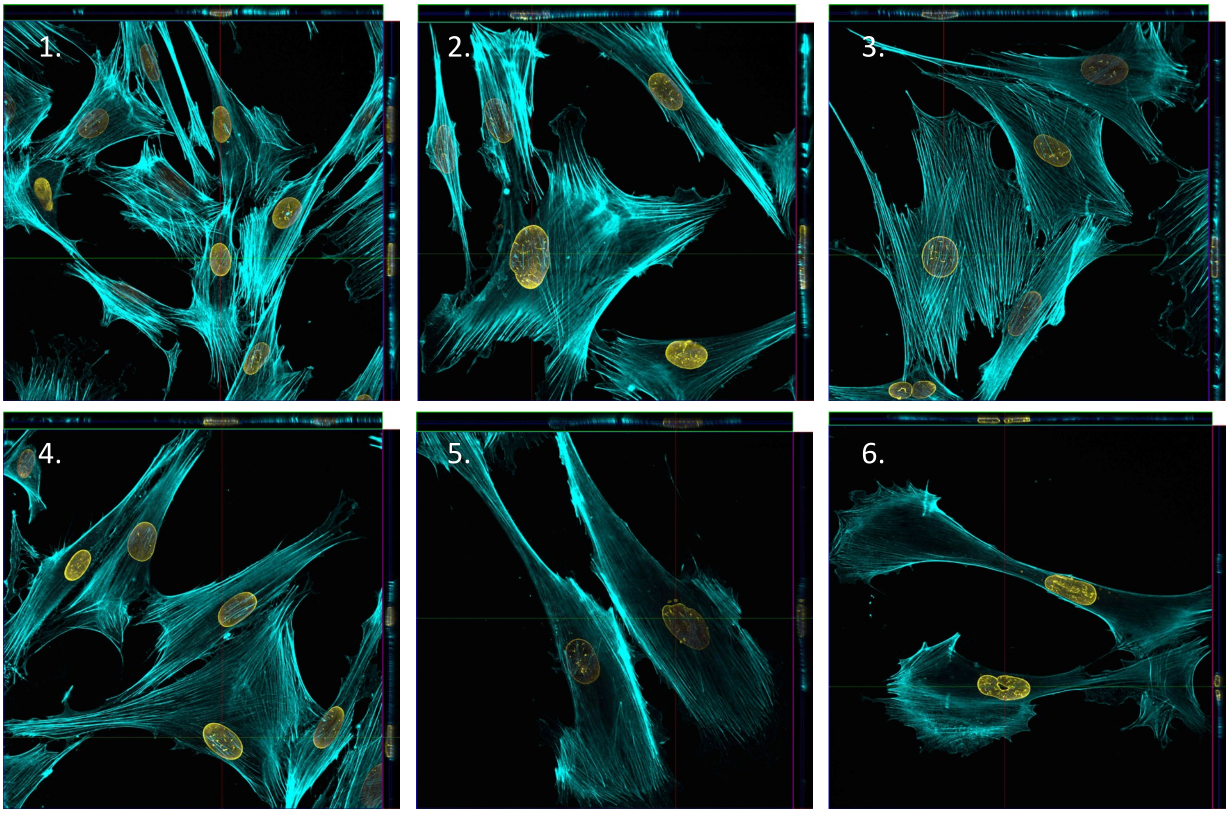

Osteogenic medium

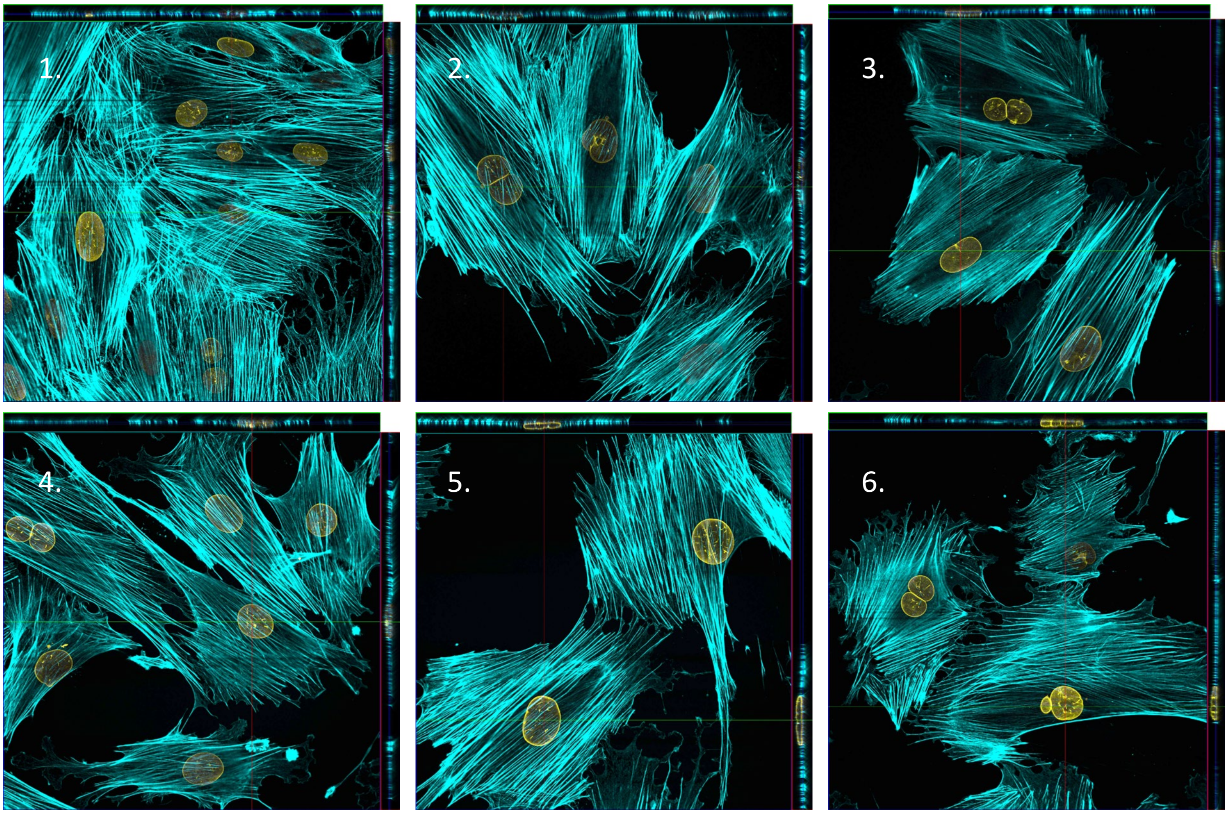

Supplementary Fig 4b Confocal microscopy of F-actin and Arp4 comparing control (MEM) and cells treated with osteoblast differentiating medium. Osteoblast cells have a highly developed F-actin structure extending extends throughout the cell, while nuclear actin is not appreciably different. Arp4 is similar between cell types.

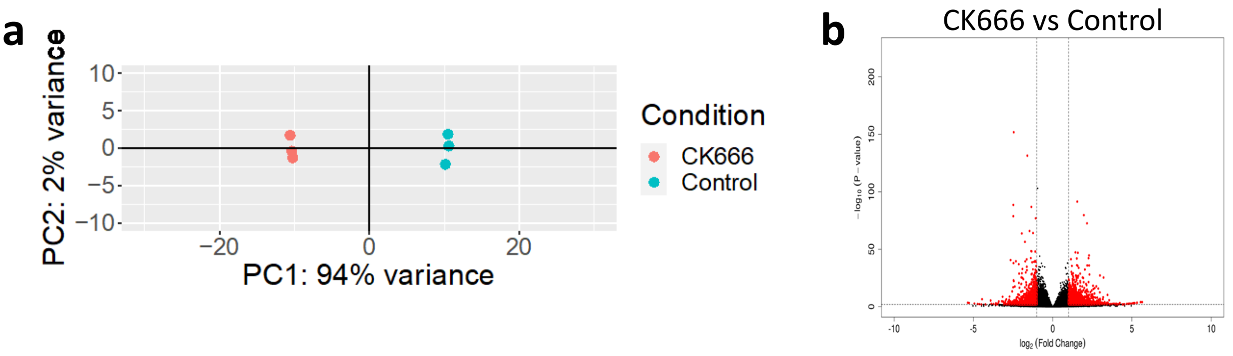

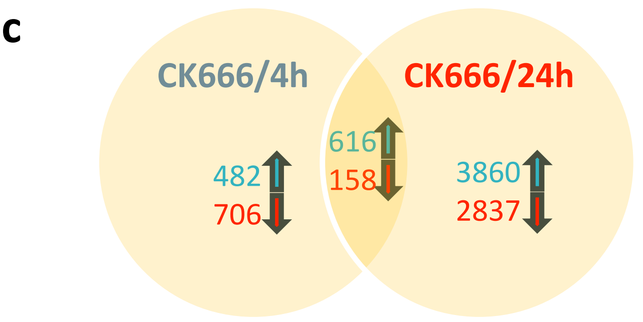

d

Pathway	CK666 (4 hours) Increased ATAC	CK666 (4 hours) Decreased ATAC
Adipogenesis	4.35471133	
UV Response Dn		1.30304192
Epithelial Mesenchymal Transition		12.0454854
Apoptosis	1.52302355	
Inflammatory Response		1.33739248
Protein Secretion		1.91890923
Apical Junction		2.24824299
TNF-alpha Signaling via NF-kB	2.01941471	1.91912175
Hypoxia		3.05794331
Myogenesis		1.6685293

Supplementary Figure 5: Chromatin accessibility altered by CK666 at 4 hours in bone marrow mesenchymal stem cells (MSCs). (A) PCA shows CK666 (red) alters chromatin accessibility even at 4 hours when compared to the baseline control state (green). (B) Addition of CK666 results in slightly more regions with significantly (p-value < 0.01) increased accessibility than regions with decreased accessibility. There are fewer significantly altered regions at 4 hours than at 24 hours (Figure 1B). (C) Many regions similarly show significant increases and decreases at 4 hours and 24 hours. (D) Pathways enriched at 4 hours highly overlap those enriched at 24 hours in the same directions (blue, red).

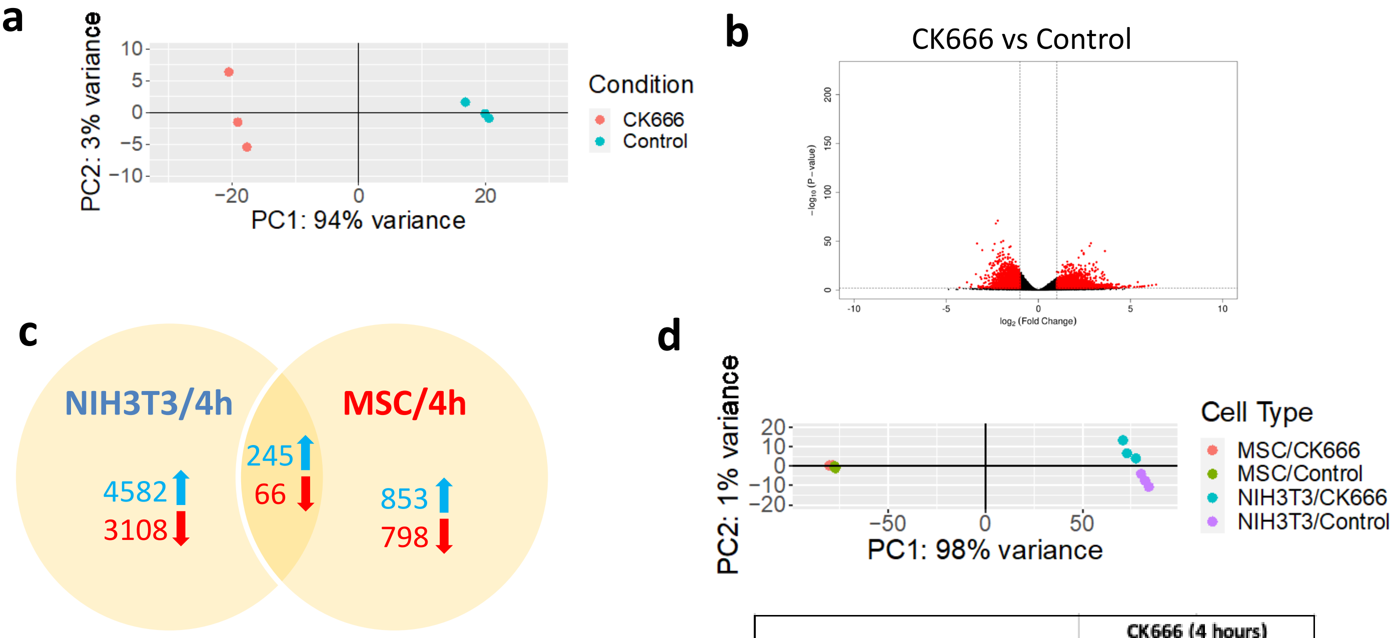

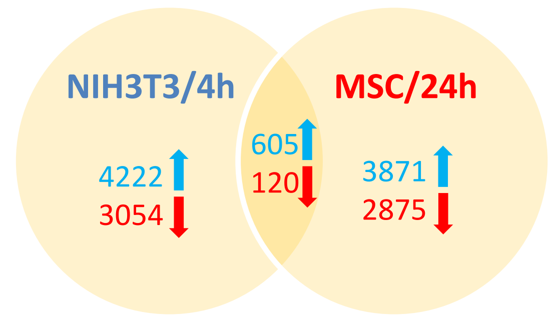

e

	CK666 (4 hours)	
	Increased	Decreased
Pathway	ATAC	ATAC
IL-2/STAT5 Signaling	3.63227909	
KRAS Signaling Up		6.45918877
UV Response Dn	3.17796503	4.23068634
Estrogen Response Early	2.23884055	
Epithelial Mesenchymal Transition	2.43246709	1.66858287
Protein Secretion		1.97814056
TNF-alpha Signaling via NF-kB	4.68241302	1.66858287
Hypoxia	2.43246709	
mTORC1 Signaling	2.04429926	
Hedgehog Signaling		1.66858287
Bile Acid Metabolism	1.71907079	

Supplementary Figure 6: Chromatin accessibility altered by CK666 at 4 hours in the NIH3T3 embryonic fibroblast cell line. (A) PCA shows CK666 (red) altered chromatin accessibility at 4 hours in NIH3T3 cells clearly separates samples when compared to the baseline control state (green). (B) Addition of CK666 results in more regions with significantly (p-value < 0.01) altered accessibility compared to MSCs at 4 hours (Figure S4B). (C) Most regions with significant changes in chromatin accessibility in NIH3T3 cells at 4 hours are distinct from changes in MSCs at 4 hours and 24 hours. There is a greater percentage of overlap between MSCs at two distinct time points, 4 and 24 hours (Figure S4C). (D) PCA plot shows that the chromatin profiles of MSCs and NIH3T3 in the baseline controls are highly distinct, especially relative to the changes induced by CK666 at 4 hours in either cell type. (E) Pathways enriched at 4 hours still highly overlap those enriched for MSCs at 24 hours in the same directions (blue, red).
