# Supplementary material for: Nuclear actin structure regulates chromatin accessibility
Source: Nat Commun. 2024 May 15;15:4095. doi: 10.1038/s41467-024-48580-y (PMC11096319; doi:10.1038/s41467-024-48580-y)
Supplement: Supplementary file 3 — Description of Additional Supplementary Files [file 41467_2024_48580_MOESM3_ESM.pdf]

Description of Additional Supplementary Files

File Name: Supplementary Data 1
Description: Regions of differential chromatin accessibility between MSC cells with CK666 added vs control cells (DESeq2 using Wald Test; adjusted p-values calculated using the Benjamini-Hochberg correction).

File Name: Supplementary Data 2
Description: Regions of differential chromatin accessibility between MSC cells with CytoD added vs control cells (DESeq2 using Wald Test; adjusted p-values calculated using the Benjamini-Hochberg correction).

File Name: Supplementary Data 3
Description: Differentially expressed genes between MSC cells with CK666 added vs control cells (DESeq2 using Wald Test; adjusted p-values calculated using the Benjamini-Hochberg correction).

File Name: Supplementary Data 4
Description: Differentially expressed genes between MSC cells with CytoD added vs control cells (DESeq2 using Wald Test; adjusted p-values calculated using the Benjamini-Hochberg correction).

File Name: Supplementary Data 5
Description: Regions of differential chromatin accessibility between MSC cells with siArp4 added vs control cells (DESeq2 using Wald Test; adjusted p-values calculated using the Benjamini-Hochberg correction).

File Name: Supplementary Data 6
Description: Regions of differential chromatin accessibility between MSC cells with siArp4 and CK666 added vs control cells (DESeq2 using Wald Test; adjusted p-values calculated using the Benjamini-Hochberg correction).

File Name: Supplementary Data 7
Description: Differentially expressed genes between MSC cells with siArp4 added vs control cells (DESeq2 using Wald Test; adjusted p-values calculated using the Benjamini-Hochberg correction).

File Name: Supplementary Data 8
Description: Differentially expressed genes between MSC cells with siArp4 and CK666 added vs control cells (DESeq2 using Wald Test; adjusted p-values calculated using the Benjamini-Hochberg correction).

File Name: Supplementary Data 9
Description: Regions of differential chromatin accessibility between MSC cells with CK666 added vs control cells, 4 hour time point (DESeq2 using Wald Test; adjusted p-values calculated using the Benjamini-Hochberg correction).

File Name: Supplementary Data 10
Description: Regions of differential chromatin accessibility between NIH3T3 cells with CK666 added vs control cells, 4 hour time point (DESeq2 using Wald Test; adjusted p-values calculated using the Benjamini-Hochberg correction).
